# Anaphylaxis as an Unusual Cause of Shock in the Trauma Setting: A Case Report

**DOI:** 10.7759/cureus.64696

**Published:** 2024-07-16

**Authors:** Williams Luciano López-Vidal, Maricela Fernanda Enriquez-Montes, Daniel A Meza-Martinez, Luis M Gallardo-Cantua, Marco A Hernandez-Guedea

**Affiliations:** 1 Emergency Shock Trauma Department, Hospital Universitario Dr. José Eleuterio Gonzalez, Monterrey, MEX; 2 General Surgery, Instituto Mexicano del Seguro Social, Hospital General de Zona No. 33, Monterrey, MEX

**Keywords:** advanced trauma life support, emergency medicine and trauma, life threatening anaphylaxis, drug-reaction, nsaid allergy

## Abstract

Anaphylactic shock is the most severe form of an acute systemic allergic reaction and can be potentially lethal if left untreated. Here, we present the case of a 51-year-old male with no significant medical history, who arrived at our hospital's emergency trauma bay following a motor vehicle accident caused by a sudden onset of malaise while driving. Upon arrival, the patient's airway was patent, but he reported a sensation of a foreign body in his larynx. He also had an oxygen saturation of 88%, although no abnormal breath sounds were auscultated. The patient was also hypotensive and tachycardic, with no favorable response after crystalloid administration. He had no neurological alterations but was diaphoretic, with hives spreading across his trunk and all four extremities. Upon further interrogation, we identified that he had consumed diclofenac, a non-steroidal anti-inflammatory drug (NSAID), 45 minutes before the driving incident. Prompt recognition and management of the anaphylactic shock were initiated alongside the assessment and treatment of the traumatic injuries. This case highlights the importance of considering unusual causes of shock in trauma patients. It underscores the need for a comprehensive approach to patient care in trauma settings, where multiple etiologies of shock should be considered and managed simultaneously.

## Introduction

Anaphylactic shock is the most severe clinical presentation of an acute systemic allergic reaction, potentially lethal if untreated, as it is a hypersensitivity reaction that can rapidly compromise the airway, ventilatory, and/or circulatory systems. Often associated with exposure to allergens such as food, insects, medications, and latex, early identification and appropriate management of anaphylaxis can be crucial for improving clinical outcomes [1]. Anaphylaxis can occur in unusual and challenging situations, including in traumatized patients. We describe the case of a 51-year-old patient who presented to the emergency department following a motor vehicle accident triggered by a sudden onset of malaise while driving. Despite an initial assessment ruling out severe traumatic injuries, the patient developed signs of anaphylactic shock, which were attributed to the prior ingestion of a non-steroidal anti-inflammatory drug (NSAID). This case underscores the importance of considering anaphylaxis as a differential diagnosis in traumatized patients. It highlights the need for a comprehensive evaluation and a multidisciplinary approach to managing these patients.

## Case presentation

We present the case of a 51-year-old male with no significant medical history who arrived at our hospital's emergency trauma bay following a motor vehicle accident. The accident was preceded by a sudden onset of malaise while driving, characterized by general weakness, visual disturbances, and a sudden rash on his left arm. He was involved in a frontal car crash while trying to pull off the road to seek help. After the incident, he was assisted by emergency medical services, who noted that he was able to walk out of the vehicle on his own and was then brought to our institution.

**Figure 1:**
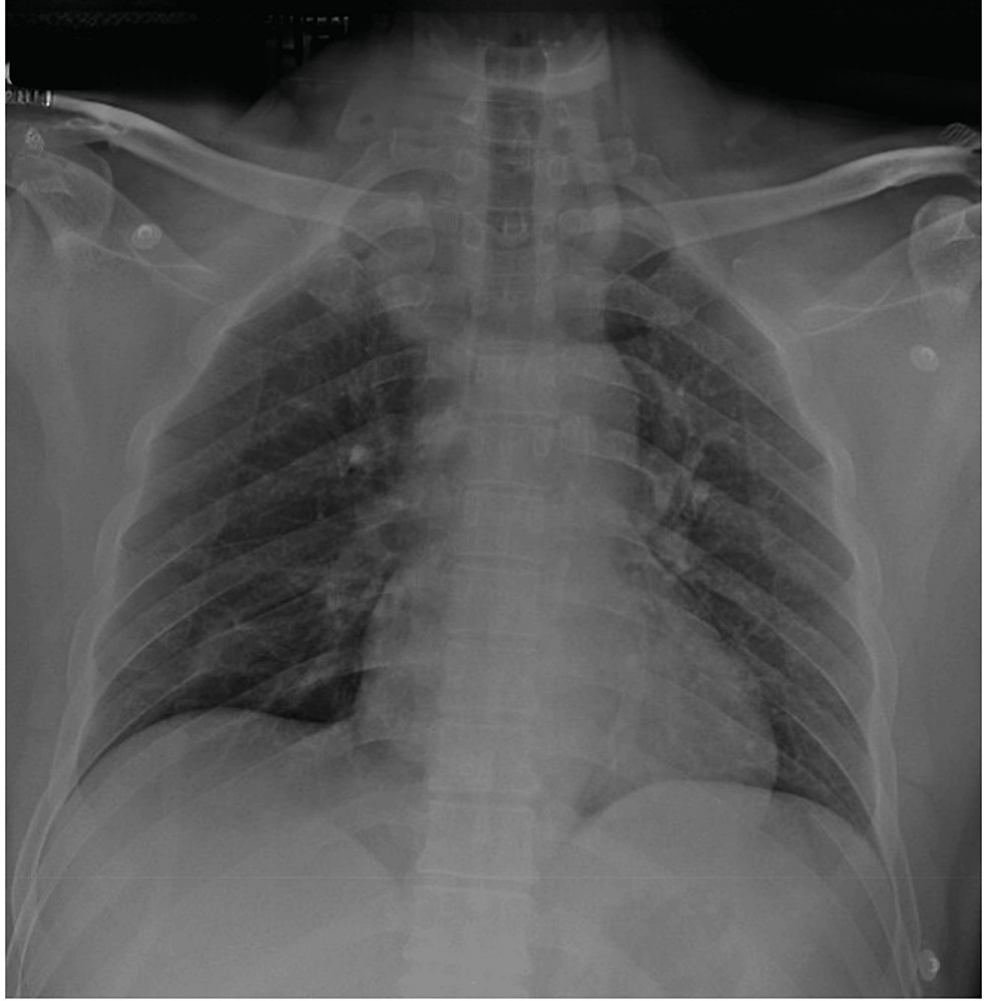
The chest X-ray demonstrated no abnormalities, with no evidence of pneumothorax, pulmonary contusion, or rib fractures.

Upon arrival at the trauma bay, the patient's airway was patent, but he reported a sensation of a foreign body in his larynx. He also had an oxygen saturation of 88%, and physical and radiographic examination of the thorax revealed no abnormalities (Figure 1).

**Figure 2:**
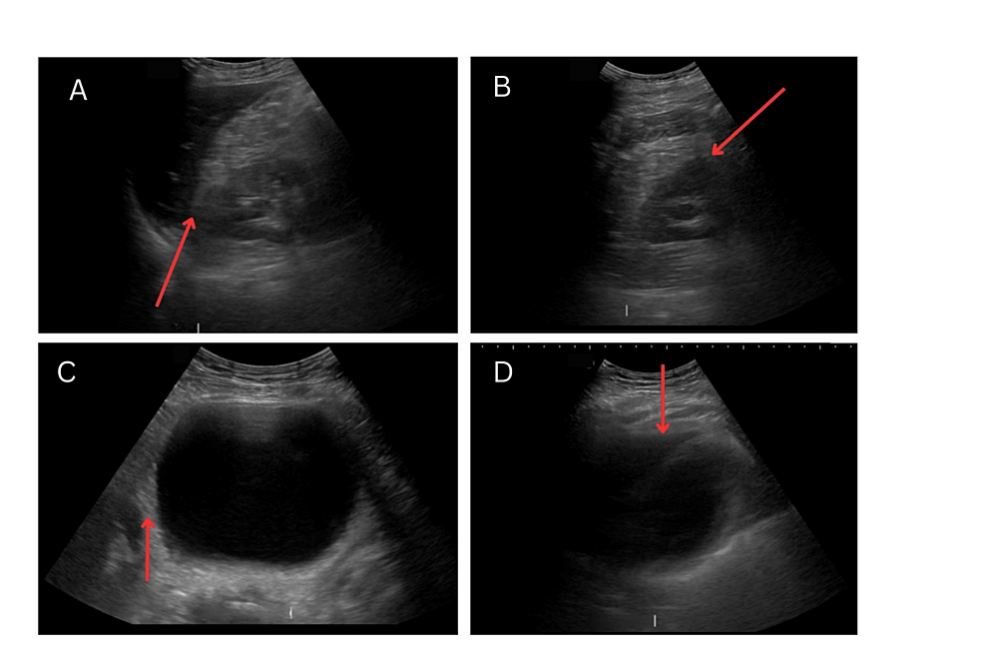
A FAST scan performed at the time of the patient's presentation revealed no abnormal findings. * A) Right flank view: Also known as the perihepatic view or Morison's pouch, showing no free fluid between the kidney and the liver. B) Left flank view: Commonly referred to as the left upper quadrant view, demonstrating no free fluid in the splenorenal interface. C) Pelvic view: Scan showing no accumulation of fluid in the rectovesical space. D) Pericardial view: Also known as the subcostal or subxiphoid view, this image demonstrates the absence of fluid in the potential space between the visceral and parietal pericardium. *: Absence of free fluid is represented by red arrows.

The patient was admitted to the emergency room for observation and further studies. Trauma protocol imaging, including plain radiographs of the chest, pelvis, and cervical spine, revealed no abnormalities, and a serial FAST exam showed no changes (Figure 2). An allergology consultation was requested, which confirmed the diagnosis of severe anaphylaxis, likely triggered by the ingestion of diclofenac.

A 24-hour observation period was deemed necessary, and antihistamines were added to the treatment regimen. The observation period passed without further incidents, and the patient was discharged home with a follow-up appointment at the outpatient allergy clinic and a prescription for an automatic epinephrine dispenser.

## Discussion

Severe anaphylaxis is characterized by an abrupt clinical reaction with rapid symptom progression and a potential compromise of the airway, breathing, or circulation, with or without skin involvement [1]. As demonstrated in the described case, the airway, breathing, and circulation can all be involved in anaphylaxis, though sometimes only some of these systems are affected. Identifying a precipitating factor is crucial for diagnosing likely cases of anaphylaxis, although it may not always be possible.

Anaphylactic reactions are often not recognized at the primary care level. Registries in the United States indicate that only about 60% of these cases are identified, and even fewer receive appropriate treatment. Only 19% of patients are administered epinephrine, and the frequency of complementary testing is also estimated to be low [2].

Clinical suspicion based on signs and symptoms should prompt early diagnosis and treatment of anaphylaxis. While available tests can be helpful, waiting for test results is not appropriate as it may delay treatment. However, in cases where there is doubt or the clinical scenario makes anaphylaxis unlikely, it can be helpful to rule out or confirm the diagnosis. Tryptase, a protease released by mast cells, is considered a gold standard test for confirming the diagnosis of anaphylaxis [3]. Unfortunately, tryptase is not available in every setting, including our institution.

Anaphylaxis can lead to cardiopulmonary arrest within 5 minutes of exposure to the allergen if left untreated, highlighting the importance of rapid and correct administration of epinephrine as a lifesaving maneuver [4]. The intramuscular application of adrenaline in the anterolateral portion of the thigh is recognized as the therapeutic standard, with intravenous infusion reserved for refractory cases [5]. Risk assessment of patients with cardiovascular disease should not delay management, as the benefits of treatment outweigh the risks in these situations [6].

Although rare, side effects can occur with the administration of epinephrine. Mild side effects may include tremors, nausea, vomiting, and palpitations, while more serious side effects such as arrhythmias and cardiac arrest can develop, although this is more likely with intravenous administration [6,7]. In our case, a life-threatening complication occurred but was properly managed without any repercussions for the patient.

Bimodal reactions can occur, with symptoms reappearing up to 12 hours after the initial presentation, which is why observation time is a safe course of action [8]. Antihistamines and steroids play a supportive role in treating anaphylaxis, as their longer acting time does not usually have an effect on the acute phase but does play a role in managing symptoms once the patient is stabilized [9,10]. The patient was discharged without any late or biphasic adverse reactions, despite having experienced a cardiac adverse effect. The use of antihistamines was not necessary. The clinical impact of this case highlights the importance of a systematic evaluation in trauma settings. A structured and sequential approach allowed for the identification of anaphylaxis, an unexpected cause of shock in this context, demonstrating the value of thorough assessment protocols to uncover even the most unusual diagnoses.

## Conclusions

This case underscores the significance of contemplating anaphylactic shock in trauma patients, even amidst inconclusive initial evaluations. Prompt recognition and treatment, including the use of epinephrine, were crucial for the patient's recovery. The favorable result emphasizes the necessity for a thorough and methodical approach to trauma care, ensuring that uncommon etiologies of shock receive appropriate consideration. 
